# Which conformation does the ABC transporter P-glycoprotein adopt in the physiological membrane environment?

**DOI:** 10.1186/2050-6511-13-S1-A68

**Published:** 2012-09-17

**Authors:** Thomas Stockner, Yaprak Dönmez, Zahida Parveen, Peter Chiba

**Affiliations:** 1 Institute of Pharmacology, Center for Physiology and Pharmacology, Medical University of Vienna, 1090 Vienna, Austria; 2 Institute of Medical Chemistry, Center for Pathobiochemistry and Genetics, Medical University of Vienna, Vienna, Austria

## Background

The human genome contains 48 members of the ABC protein family. We focus on the multidrug resistance transporter P-glycoprotein (P-gp, ABCB1), which is expressed at the blood-brain-barrier, in intestine, kidney, liver and macrophages. The first structure of an ABC exporter was from *Staphylococcus aureus* and showed a twisted architecture. The same fold was observed in MsbA, mouse P-glycoprotein and the human mitochondrial ABCB10 transporter. Although ABC exporters have now been crystallized in several conformations, uncertainty remained with respect to the physiological conformation because they seem not to be fully compatible with all biochemical evidence.

## Methods

We applied homology modeling and MD simulations to determine the equilibrium conformation of the membrane-inserted transporter to test the hypothesis whether the observed conformations might be a consequence of the crystallization procedure or conditions. We inserted the transporter model into a pre-equilibrated membrane and carried out equilibrium simulations.

## Results and conclusions

In equilibrium we observe the wings to come close, which is in compliance with experimental observations. Water becomes expelled from the hydrophobic region and the open passage between the water-filled pore and the cell exterior closes. Our results indicate that the closed conformation is energetically more favourable.

